# Engineering biocompatible TeSe_x_ nano-alloys as a versatile theranostic nanoplatform

**DOI:** 10.1093/nsr/nwaa156

**Published:** 2020-07-06

**Authors:** Xiang Ling, Zhaokui Jin, Qi Jiang, Xiaotao Wang, Bin Wei, Zhongchang Wang, Yangsen Xu, Tianye Cao, Jonathan W Engle, Weibo Cai, Chenliang Su, Qianjun He

**Affiliations:** International Collaborative Laboratory of 2D Materials for Optoelectronic Science & Technology, Engineering Technology Research Center for 2D Materials Information Functional Devices and Systems of Guangdong Province, Institute of Microscale Optoelectronics, Shenzhen University, Shenzhen 518060, China; Guangdong Provincial Key Laboratory of Biomedical Measurements and Ultrasound Imaging, National-Regional Key Technology Engineering Laboratory for Medical Ultrasound, School of Biomedical Engineering, Health Science Center, Shenzhen University, Shenzhen 518060, China; Guangdong Provincial Key Laboratory of Biomedical Measurements and Ultrasound Imaging, National-Regional Key Technology Engineering Laboratory for Medical Ultrasound, School of Biomedical Engineering, Health Science Center, Shenzhen University, Shenzhen 518060, China; International Collaborative Laboratory of 2D Materials for Optoelectronic Science & Technology, Engineering Technology Research Center for 2D Materials Information Functional Devices and Systems of Guangdong Province, Institute of Microscale Optoelectronics, Shenzhen University, Shenzhen 518060, China; Department of Quantum and Energy Materials, International Iberian Nanotechnology Laboratory (INL), Braga 4715-330, Portugal; Department of Quantum and Energy Materials, International Iberian Nanotechnology Laboratory (INL), Braga 4715-330, Portugal; International Collaborative Laboratory of 2D Materials for Optoelectronic Science & Technology, Engineering Technology Research Center for 2D Materials Information Functional Devices and Systems of Guangdong Province, Institute of Microscale Optoelectronics, Shenzhen University, Shenzhen 518060, China; Guangdong Provincial Key Laboratory of Biomedical Measurements and Ultrasound Imaging, National-Regional Key Technology Engineering Laboratory for Medical Ultrasound, School of Biomedical Engineering, Health Science Center, Shenzhen University, Shenzhen 518060, China; Departments of Radiology and Medical Physics, University of Wisconsin-Madison, Madison, WI 53705, USA; Departments of Radiology and Medical Physics, University of Wisconsin-Madison, Madison, WI 53705, USA; Departments of Radiology and Medical Physics, University of Wisconsin-Madison, Madison, WI 53705, USA; International Collaborative Laboratory of 2D Materials for Optoelectronic Science & Technology, Engineering Technology Research Center for 2D Materials Information Functional Devices and Systems of Guangdong Province, Institute of Microscale Optoelectronics, Shenzhen University, Shenzhen 518060, China; Guangdong Provincial Key Laboratory of Biomedical Measurements and Ultrasound Imaging, National-Regional Key Technology Engineering Laboratory for Medical Ultrasound, School of Biomedical Engineering, Health Science Center, Shenzhen University, Shenzhen 518060, China

**Keywords:** nanomedicine, nano-alloys, photothermal therapy, cancer nanotheranostics, biomedical imaging

## Abstract

Photothermal nanotheranostics, especially in the near infrared II (NIR-II) region, exhibits a great potential in precision and personalized medicine, owing to high tissue penetration of NIR-II light. NIR-II-photothermal nanoplatforms with high biocompatibility as well as high photothermal effect are urgently needed but rarely reported so far. Te nanomaterials possess high absorbance to NIR-II light but also exhibit high cytotoxicity, impeding their biomedical applications. In this work, the controllable incorporation of biocompatible Se into the lattice of Te nanostructures is proposed to intrinsically tune their inherent cytotoxicity and enhance their biocompatibility, developing TeSe_x_ nano-alloys as a new kind of theranostic nanoplatform. We have uncovered that the cytotoxicity of Te nanomaterials primarily derives from irreversible oxidation stress and intracellular imbalance of organization and energy, and can be eliminated by incorporating a moderate proportion of Se (x = 0.43). We have also discovered that the as-prepared TeSe_x_ nano-alloys have extraordinarily high NIR-II-photothermal conversion efficiency (77.2%), ^64^Cu coordination and computed tomography contrast capabilities, enabling high-efficacy multimodal photothermal/photoacoustic/positron emission tomography/computed tomography imaging-guided NIR-II-photothermal therapy of cancer. The proposed nano-alloying strategy provides a new route to improve the biocompatibility of biomedical nanoplatforms and endow them with versatile theranostic functions.

## Teaser

A nano-alloying strategy is proposed to completely eliminate the toxicity of Te nanomaterials and simultaneously endow them with versatile theranostic functions by Se incorporation, realizing safe, precise and high-efficacy cancer treatment by multimodal PT/PA/CT/PET imaging-guided NIR-II-photothermal therapy.

## INTRODUCTION

Nanotheranostics makes use of nanotechnology to integrate diagnostics and therapeutics, exhibiting a great potential in precision and personalized medicine [1–3]. The emergence of diverse multifunctional nanomaterials and advanced nanotechnologies unprecedentedly simulates the evolution of nanotheranostics, and enables the integration of multimodal imaging and therapeutic functions in a single theranostic nanoplatform for high-efficacy theranostics of diseases [4–6]. In engineering of theranostic nanoplatforms, biocompatibility and multifunction are two of the most important factors which need to be considered. Among various nanotheranostics, multimodal imaging-guided photothermal therapy has attracted intensive attention owing to the fact that it is less invasive and has fewer side effects compared with conventional radiotherapy and chemotherapy [7–11]. In recent years, a number of photothermal theranostic nanoagents, including noble metal nanoparticles [12–16], two-dimensional (2D) nanosheets [17–26] and organic polymer nanomaterials [27–30], have been explored for cancer treatment, but most of them only work in the NIR-I region and the candidates of theranostic nanomedicine for NIR-II-thermal imaging and therapy are quite rare. There are many photothermal theranostic nanoagents with strong absorbance ranging from NIR-I to NIR-II. However, photothermal performance of nanoagents is determined by three factors: molar extinction coefficient, photothermal conversion efficiency and allowable laser power density. Even though some nanoagents have relatively high molar extinction coefficient or considerable photothermal conversion efficiency in the NIR-II window, their photothermal performances in the NIR-II window are not as good as those in the NIR-I window because of lower allowable laser power density [31]. However, it is true that compared with NIR-I light, NIR-II light possesses some intrinsic advantages in lesser photo-scattering and higher maximum permissible exposure (MPE), consequently exhibiting higher tissue penetration depth with less background interference and higher spatial resolution, and allowing tissue illustration at a relatively higher power density of laser (1.0 W cm^−2^ for NIR-II versus 0.3 W cm^−2^ for NIR-I) [29]. Therefore, to develop biocompatible NIR-II-photothermal nanoplatforms with versatile imaging functions is significant to precision cancer theranostics but challenging.

**Scheme 1.**
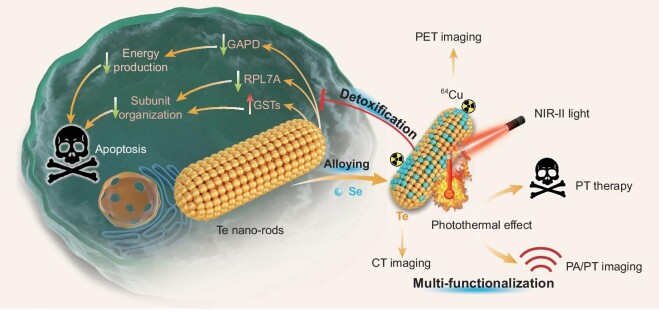
Schematic illustration of TeSe_x_ alloying strategy and mechanisms for detoxification and theranostic multi-functionalization. Several main advances are achieved: (i) advanced TeSe_x_ nano-alloys are facilely constructed to intrinsically eliminate the inherent toxicity of Te nanomaterials by the moderate incorporation of biocompatible Se; (ii) advanced mechanisms for Te nanomaterial toxification and TeSe_x_ alloying detoxification are uncovered; (iii) advanced theranostic performances with extraordinarily high NIR-II-photothermal efficiency and multimodal PT/PA/CT/PET imaging capability are achieved by nano-alloying.

Both selenium (Se) and tellurium (Te) belong to the chalcogen elements, and their nanomaterials exhibit some unique semi-conductive features [32–36]. Te nanoneedles and nanosheets have an extremely narrow band gap (about 0.35 eV) and a strong absorbance in the NIR-II region in support of NIR-II-photothermal therapy and imaging, but also demonstrate high cytotoxicity and poor biocompatibility owing to their strong reducibility, restricting their biomedical applications [37–41]. By comparison, Se is an essential element for human beings and the selenizing can eliminate the cytotoxicity of many metals such as Cd and Cu [42,43]. Therefore, we hypothesize that controllable incorporation of biocompatible Se into the lattice of Te nanostructures for construction of TeSe_x_ nano-alloys could intrinsically tune the inherent cytotoxicity of Te nanomaterials, enhance the biocompatibility of Te nanomaterials and extend their functions for biomedical applications. In this work, we synthesize a series of TeSe_x_ nano-alloys with different Se incorporating proportions, and investigate their biocompatibility and develop their theranostic functions. We have discovered that the toxicity of Te nanomaterials mainly comes from irreversible oxidation stress and intracellular imbalance of organization and energy, which is exterminated by the nano-alloying by incorporating a moderate proportion of Se (x = 0.43) (Scheme 1). The synthesized TeSe_x_ nano-alloy (x = 0.43) exhibits extraordinarily high NIR-II-photothermal conversion efficiency (77.2%), ^64^Cu coordination and computed tomography (CT) contrast capabilities, enabling high-efficacy photothermal therapy of cancer under the guidance of multimodal photothermal (PT)/photoacoustic (PA)/positron emission tomography (PET)/CT imaging (Scheme 1).

## RESULTS AND DISCUSSION

### Synthesis and characterization of TeSe_x_ nano-alloys

**Figure 1.**
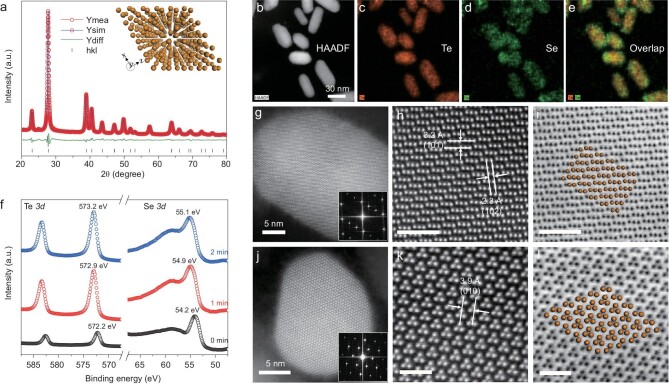
The structure, morphology and chemical composition of TeSe_x_ nano-alloys (TS3). (a) Measured (red line) and simulated (blue line) XRD patterns, and differentiated profiles (green line) between them obtained from the Rietveld refinement of TS3 using *P3_1_21* space group with hexagonal unit cell, where the inset is the perspective view of the simulated crystal structure of TeSe_x_ nano-alloys (the atoms represent either Te or Se). HAADF-STEM (b) and energy dispersive spectrum (EDS) elemental-mapping images (c−e) of TeSe_x_ nano-alloys. (f) Depth profiling XPS spectra of TeSe_x_ nano-alloys exposed to Ar^+^ for 0, 1 and 2 min. Atomically resolved HAADF-STEM images acquired along the *a*-axis direction (g) and *c*-axis direction (j), with more detailed views (h, k) and corresponding BF-STEM images (i, l). The scale bars in (h), (i), (k) and (l) correspond to 2 nm, 1 nm, 2 nm and 1 nm, respectively.

A series of rod-like TeSe_x_ nano-alloys with various ratios of Se/Te and length/diameter were synthesized by a facile co-precipitation method. Tellurite and selenite were reduced simultaneously by hydrazine to form TeSe_x_ nano-alloys, and the Se contents were adjusted by tuning the molar ratio of tellurite to selenite (Supplementary Table S1). TeSe_x_ nano-alloys with Se/Te precursor molar ratios of 1 : 3, 2 : 3, 1 : 1 and 3 : 2 were named as TS1, TS2, TS3 and TS4, respectively. To ascertain the crystal structure of the as-prepared TeSe_x_ nanomaterials, X-ray diffraction (XRD) characterization was conducted (Fig. 1a and Supplementary Fig. S1). XRD patterns were further refined using the total pattern solution (TOPAS) Rietveld crystal-structure refinement software (Fig. 1a and Supplementary Fig. S2). The refinement results suggested the formation of Te-Se alloys, which were crystallized in a rhombohedral structure with *P3_1_21* space group. The refined structure of typical TS3 nano-alloy was further investigated by high-resolution transmission electron microscope (TEM). In a high-angle annular dark field (HAADF) image and the corresponding elemental mapping in Fig. 1b–e, Te and Se were dispersed throughout the whole rod-like TeSe_x_ nano-alloys, and no core-shell structure can be obviously observed. To study the radial elemental distribution of the as-prepared TeSe_x_ nano-alloys, depth profiling X-ray photoelectron spectroscopy (XPS) analysis was conducted on sample TS3 which was exposed to Ar^+^ for 0, 1 and 2 min. Te *3d* and Se *3d* XPS spectra of TS3 in Fig. 1f revealed that there were only Te (0) and Se (0) in the TeSe_x_ nano-alloy, and the binding energy of Te *3d* and Se *3d* gradually decreased with the increase of Ar^+^ etching time. Accordingly, the atomic ratio of Se to Te obtained from XPS (Supplementary Table S2) decreased gradually, suggesting the gradient increase of Se content from inside to outside in support of the formation of TeSe_x_ nano-alloy [44]. To confirm the atomic structure of TeSe_x_ nano-alloy, atomically resolved HAADF-scanning transmission electron microscopy (STEM) was conducted. Figure 1g–i and Fig. 1j–l showed the simultaneously recorded HAADF and bright-field (BF) STEM images acquired along *a*-axis and *c*-axis, respectively. The observed atomic crystal structure from STEM was in high accordance with the simulated one from XRD refinements (yellow spheres in Fig. 1i and l). Besides, no megascopic difference between Te and Se can be observed, pinpointing that Se and Te were thoroughly miscible in each other and formed homogenous trigonal-system TeSe_x_ nano-alloy.

The compositions of a series of TeSe_x_ nano-alloys were measured by the inductively coupled plasma optical emission spectrometry (ICP-OES). As shown in Supplementary Table S1, the chemical structures of TS1, TS2, TS3 and TS4 were Te_0.82_Se_0.18_, Te_0.75_Se_0.25_, Te_0.7_Se_0.3_ and Te_0.67_Se_0.33_, respectively. By varying the amounts of Se and Te precursors, the molar fraction of Se in TeSe_x_ nano-alloys could be controllably tailored. XRD patterns of TS1, TS2, TS3 and TS4 in Supplementary Fig. S1 showed that the diffraction peaks were well matched with the standard ones of Te (JCPDS card number 36–1452) in the absence of impurity. In contrast to the reported core−shell Te@Se nanowires [45] and Se-coated Te nanoheterojunctions [46], no characteristic XRD peaks of Se were observed in TeSe_x_ nano-alloys in this work. It is worth carefully noticing that the finely identified TeSe_x_ nano-alloying structure is easily mistaken for core-shell structure and heterojunction, possibly attributed to improper sampling, characterization and analysis. As the content of Se in TeSe_x_ nano-alloys increased, all the diffraction peaks shifted slightly towards high-angle direction, suggesting the decrease of the interlayer distances which agreed with the variation of *c*/*a* obtained from the Rietveld refinement results (Supplementary Table S3). XPS analysis (Supplementary Fig. S3) displayed that with the increase of the amount of Se, Te *3d* and Se *3d* peaks of TeSe_x_ nano-alloys shifted towards lower binding energy, which was also induced by the alloying formation between Te and Se. TEM images in Supplementary Fig. S4 indicated that the diameter and length of rod-like TeSe_x_ nano-alloys decreased with the increase of Se incorporation amount, while their morphologies remained nearly unchanged, suggesting that the incorporation of Se inhibited the growth of Te nano-rods. The particle size of synthesized TeSe_x_ nano-alloys was less than 100 nm in favor of passive targeting accumulation in tumor by the enhanced permeability and retention (EPR) effect.

### Evolution of biocompatibility and photothermal properties of TeSe_x_ nano-alloys

**Figure 2.**
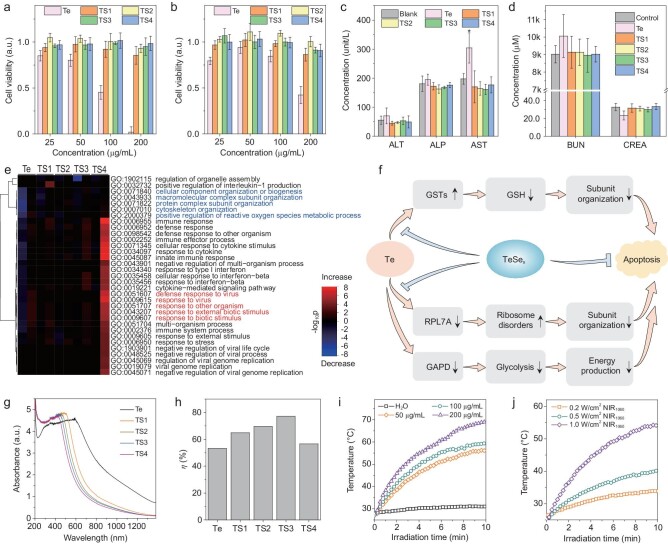
Biocompatibility and photothermal properties of TeSe_x_ nano-alloys. Cytotoxicity (a) and cell proliferation (b) of 4T1 cells incubated with TeSe_x_ nano-alloys at varied particle concentrations. Haematological indexes of liver (c) and kidney (d) functions of the mice with intravenous administration of TeSe_x_ nano-alloys. (e) Heat map showing scaled expression values of the differentially expressed genes for TeSe_x_ nano-alloys vs. blank control comparison. (f) Schematic illustration of significant pathways of Te toxification and TeSe_x_ detoxification. (g) Absorption spectra of TeSe_x_ nano-alloys (200 μg mL^−1^). (h) Calculated NIR-II-photothermal conversion efficiencies of Te nano-rods and TeSe_x_ nano-alloys with 1060 nm laser. NIR-II-photothermal curves of TeSe_x_ solutions under the irradiation of 1060 nm laser at varied particle concentrations (50, 100 and 200 μg mL^−1^) at the same laser power density of 1 W cm^−2^ (i), and at different power densities (0.2, 0.5 and 1 W cm^−2^) at the same particle concentration of 100 μg mL^−1^ (j). *P* values in c were calculated by two-tailed Student's *t*-test (^*^P < 0.05) by comparing with the blank control group.

The biocompatibility of nanomedicines is vitally important to their biomedical application. Here we evaluated the biocompatibility of TeSe_x_ nano-alloys and checked the effect of the Se incorporation amount. Two cell lines (breast 4T1 cells and liver L-O2 cells) were employed for *in vitro* cytotoxicity assay. In Fig. 2a and b and Supplementary Fig. S5, when incubated with different samples at varied concentrations for 24 h, Te nano-rods showed obvious inhibition effect to the growth and proliferation of both 4T1 and L-O2 cells, even at the low concentration of 25 μg mL^−1^. In comparison, all the investigated TeSe_x_ nano-alloys did not exhibit significant cytotoxicity at the concentration of 25−100 μg mL^−1^. At the high concentration of 200 μg mL^−1^, TS1 and TS2 with relatively lower Se incorporation amounts still can inhibit cell growth to a certain extent, while TS3 and TS4 did not (Fig. 2b). *In vivo* toxicity of TeSe_x_ nano-alloys was further investigated. After intravenous injection with TeSe_x_ nano-alloys at the higher dose of 50 mg kg^−1^ for one week, all the treated mice were alive and well, and their blood samples were taken from the orbital sinus to investigate the toxicity of TeSe_x_ nano-alloys. From the standard blood biochemical indexes in Fig. 2c and d, the concentrations of aspartate transaminase (AST) and creatinine (CREA) in the Te-treated group were remarkably higher and lower than that of the blank control group, respectively, suggesting that Te nano-rods caused distinct damage to liver and kidney functions. By comparison, all the investigated TeSe_x_ nano-alloys did not demonstrate visible toxicity to liver and kidney. These *in vitro* and *in vivo* toxicity results therefore suggested that the incorporation of Se into Te nano-rods at a relatively high amount can effectively reduce their toxicity. In addition, different from TeSe nanoheterojunctions [46], Te nano-rods synthesized in this work can degrade by only 1.4% in water after immersion for 8 months, and nano-alloying of TeSe_x_ inhibited the

degradation of Te nano-rods remarkably (Supplementary Table S4) in support of depressed toxicity.

To understand the mechanisms of the toxicity of Te nano-rods and alloying detoxification, gene expression studies were performed by RNA sequencing (RNA-Seq) which allowed quantitative measurement of expression levels of genes in 4T1 cells from six groups with different treatments (blank control, Te, TS1, TS2, TS3 and TS4 at 200 μg mL^−1^). We first screened the differentially expressed genes (DEGs) between TeSe_x_ nano-alloys and blank control to characterize the functional consequences of gene expression changes induced by TeSe_x_ nano-alloys. As shown in Supplementary Figs S6 and S7, there were 201 (Te), 172 (TS1), 199 (TS2), 198 (TS3) and 189 (TS4) DEGs between Te/TeSe_x_ and blank control, respectively, where 110 (Te), 71 (TS1), 101 (TS2), 80 (TS3) and 114 (TS4) genes were up-regulated by Te/TeSe_x_, respectively. The heat map in Supplementary Fig. S8 clearly shows that the negative effect of Te on gene expression was significantly attenuated by the incorporation of Se. To further identify the functions of these DEGs, we performed gene ontology (GO) analysis. In Fig. 2e, several genes, involving subunit organization and positive regulation of reactive oxygen species (ROS) metabolic process, were remarkably down-regulated by Te nano-rods, but very slightly affected by TeSe_x_ nano-alloys, especially TS3 and TS4 with higher Se incorporation amounts. Furthermore, defense response was provoked by Te nano-rods and can also be avoided to a certain extent by TS1, TS2 and TS3 with relatively lower Se incorporation amounts. However, excessive Se incorporation for TS4 caused the strongest defense response. These GO results indicated that Te nano-rods disturbed the normal metabolic process of ROS and thus caused oxidative stress and damage to subunit organization. The moderate incorporation of Se into Te nano-rods (such as TS3) can recover the normal metabolic process of ROS and avoid damage to subunits, thereby greatly reducing the toxicity of Te nano-rods. But the defense response from overhigh incorporation of Se (such as TS4) would possibly cause toxicity. To further identify the pathways of Te toxification and TeSe_x_ detoxification, we performed the pathway analysis of DEGs based on the KEGG (Kyoto Encyclopedia of Genes and Genomes) database. As summarized in Fig. 2f, and Supplementary Tables S5 and S6, Te nano-rods positively stimulated the drug metabolism pathway, which was similar to the response of cells to many toxic substances (Supplementary Fig. S9a). Furthermore, Te nano-rods significantly promoted the metabolism of glutathione (GSH) by up-regulating glutathione S-transferase (GSTs), resulting in the decrease of intracellular GSH level and thus oxidative stress to impair subunit organization, as illustrated in Fig. 2f. Moreover, Te nano-rods also significantly caused ribosome disorders and inhibited glycolysis by suppressing RPL7A and GAPD, consequentially causing subunit organization dysfunction and reduced energy production, as illustrated in Fig. 2f. The increased levels of GSTs protein expression and decreased levels of RPL7A and GAPD protein expression in Te evidently confirmed the associated GSH metabolism and energy production. In contrast, the TS3 got rid of the negative effects on GSTs, RPL7A and GAPD (Supplementary Fig. S10). Therefore, the Se incorporation into Te nano-rods to form TeSe_x_ nano-alloys got rid of the negative effects on GSTs, RPL7A and GAPD (Supplementary Tables S5 and S6 and Fig. S10), suppressing the cytotoxicity of Te (Fig. 2f). The GSH depletion of Te nanomaterials by surface coordination between Te and hydrosulfide group is generally thought to be the main reason for their toxicity [47]. Indeed, this work also found that Te nano-rods could adsorb GSH but TeSe_x_ nano-alloys almost not (Supplementary Fig. S11). Additionally, this work discovered that Te can also reduce GSH by up-regulating GSTs, and also uncovered other pathways involving ribosome and glycolysis for the first time. The identified mechanisms for Te toxification and TeSe_x_ detoxification in this work would greatly favor deep understanding of the origination of Te nanomaterials toxicity and also provide a strategy for developing biocompatible Te-based nanomaterials for biomedical applications.

After detoxification, the NIR-II-photothermal effect of TeSe_x_ nano-alloys was evaluated. As shown by UV–VIS–NIR spectra in Fig. 2g, all the investigated TeSe_x_ nano-alloys (TS1–TS4) had distinct NIR-II light absorption. The Se incorporation led to the blue shift of absorption spectra and the reduction in the NIR-II absorbance of Te nano-rods, but remarkably enhanced their NIR-II-photothermal conversion efficiencies. As shown in Fig. 2h and Supplementary Fig. S12, the NIR-II-photothermal conversion efficiency of TeSe_x_ nano-alloys gradually increased and then decreased with the increase of Se incorporation amount. TS3 exhibited the highest NIR-II-photothermal conversion efficiency (*η*) of 77.2% under the irradiation of a 1060 nm laser, which is much higher than that of Te nano-rods (53.3%) and other reported NIR-II-photothermal nanomaterials such as Au nanostar@MOF (48.5%) [48], Nb_2_C nanosheet (46.7%) [19] and Pt spiral (52.5%) [49]. Moreover, the aqueous solution of TS3 nano-alloy was exposed to the 1060 nm laser at varied laser power densities (0.2, 0.5 and 1.0 W cm^–2^) and at different particle concentrations (50, 100 and 200 μg mL^–1^) to investigate its NIR-II-photothermal effect. As shown in Fig. 2i and j, the NIR-II-photothermal effect of TeSe_x_ nano-alloy was positively related to both the power density of the laser and the concentration of TeSe_x_ nano-alloy. Typically, the temperature of the TeSe_x_ solution containing 200 μg mL^–1^ TS3 rose by 36.2°C after 7 min of 1060 nm laser irradiation at 1.0 W cm^–2^ in great support of thermal therapy of cancer. The NIR-II-photothermal stability of TS3 was further investigated for five laser on/off cycles. As shown in Supplementary Fig. S13, a temperature change of 36.6°C was achieved and did not show significant deterioration during five-cycle irradiation, suggesting that TeSe_x_ nano-alloy had high NIR-II-photothermal stability. From the above results, TeSe_x_ nano-alloys with moderate Se incorporation demonstrated highest comprehensive performances including good biocompatibility and high NIR-II-photothermal efficiency, and was therefore chosen as a theranostic platform to execute the following evaluation of theranostic performances. In addition, we further measured photothermal performances of TeSe_x_ nano-alloys using a 808 nm laser and compared them with the use of a 1060 nm laser. As in Fig. 2 and Supplementary Fig. S14, it could be found that NIR-II-photothermal conversion efficiency of TS3 nano-alloys (77.2% for 1060 nm) was higher than that in the NIR-I window (62.3% for 808 nm, Supplementary Fig. S14b). Although TeSe_x_ nano-alloys had higher extinction coefficient at 808 nm than at 1060 nm (Supplementary Fig. S15), they still exhibited higher photothermal performance at 1060 nm (Fig. 2, Supplementary Figs S14 and S16) owing to higher photothermal efficiencies. Therefore, at the same laser power density, a 1060 nm laser should have a higher tissue penetration depth than a 808 nm laser, and PAI performance of TS3 nano-alloys in the NIR-II window could be better than that in the NIR-I window [27], implying the possibility of using TS3 nano-alloys for photoacoustic imaging in both NIR-I and NIR-II windows. In addition, we have executed the measurement of singlet oxygen yield under NIR irradiation, and found that no distinct singlet oxygen was generated by TeSe_x_ nano-alloys (Supplementary Fig. S17), possibly because most of the photo energy had been converted to heat.

### *In vivo* multimodal imaging performances of TeSe_x_ nano-alloys

**Figure 3.**
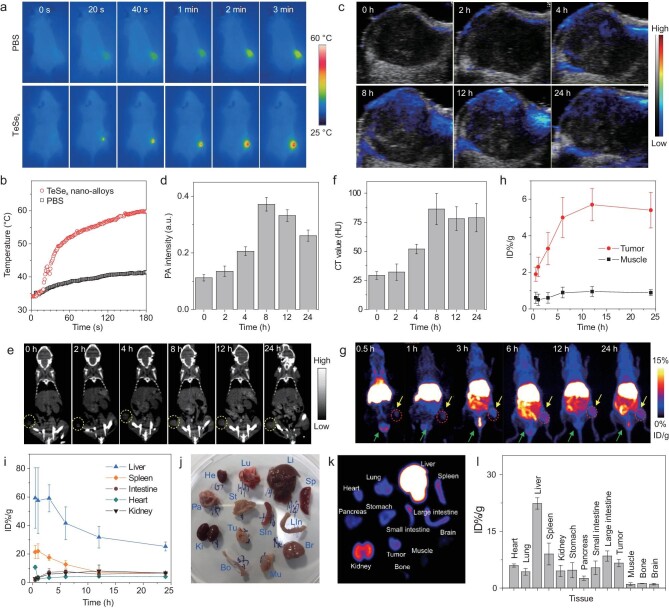
*In vivo* multimodal imaging performances of TeSe_x_ nano-alloys (TS3). (a) *In vivo* photothermal imaging tracking of one 4T1 tumor-bearing mouse under the 1060 nm laser irradiation after intravenous injection with PBS or TeSe_x_ nano-alloys, and (b) the temperature change at the tumor site. (c) *In vivo* PA images and (d) PA signal intensity change at the tumor site of one 4T1 tumor-bearing mouse before and after intravenous injection of TeSe_x_ nano-alloys. (e) *In vivo* CT images and (f) CT signal intensity change at the tumor site of one 4T1 tumor-bearing mouse before and after intravenous injection of TeSe_x_ nano-alloys. (g) *In vivo* PET images of one 4T1 tumor-bearing mouse obtained at different time points post injection of ^64^Cu-labeled TeSe_x_ nano-alloys. (h, i) Quantitative biodistribution obtained from ROI (region of interest) analysis of PET images. *Ex vivo* digital (j) and PET (k) images and the corresponding biodistribution (l) of main organs after injection for 24 h.

Inherent imaging functions of theranostic nanoplatforms are very helpful for precision medicine. Especially, multimodal imaging with complementary advantages can be used to accurately guide cancer therapy. Based on the unique properties of TeSe_x_ nano-alloys in photothermal conversion, surface incorporation/coordination and high density (high atomic number), we tried to uncover multimodal PT/PA/PET/CT imaging performances of TeSe_x_ nano-alloys with the 4T1 tumor-bearing mice model. As to PT imaging, TeSe_x_ nano-alloys (100 μL of TS3 at 2 mg mL^–1^) were intravenously injected into mice, when tumors grew up to 100 mm^3^, followed by 1060 nm laser irradiation (1 W cm^–2^, 5 min) after 8 h post injection. The TeSe_x_ group had a remarkably higher increase of temperature in the irradiated tumor site compared with the phosphate buffer saline (PBS) control group (Fig. 3a and b). After 1 min NIR-II light irradiation, the increases of temperature in the TeSe_x_ and PBS groups were 17.6°C and 4.2°C, respectively, suggesting that the irradiated tissue itself had low NIR-II-photothermal effect but TeSe_x_ nano-alloys effectively accumulated in the tumor in a passive targeting way and exhibited high NIR-II-photothermal effect owing to high NIR-II-photothermal conversion efficiency (Fig. 2h). Based on NIR-II-photothermal effect, the PA imaging (PAI) performance of TeSe_x_ nano-alloys was further evaluated *in vitro* and *in vivo* on the 4T1 tumor-bearing mice model. TeSe_x_ nano-alloys exhibited a high photoacoustic coefficient of 0.109 mL mg^−1^ at 810 nm (Supplementary Fig. S18). As shown in Fig. 3c, the tumor itself displayed relatively low PA signal before injection. After intravenous injection of TeSe_x_ nano-alloys (TS3), the intratumoral PA signal intensity gradually augmented over time and reached the maximum value at about 8 h post injection (Fig. 3d), suggesting efficient intratumoral accumulation of TeSe_x_ nano-alloys in accordance with the above-mentioned PT results.

Leveraging the virtue of large X-ray absorption coefficient of high atomic number elements for CT contrast and the strong affinity of chalcogen to transitional metal ions for PET imaging [50,51], we anticipated that TeSe_x_ nano-alloys could impart the quantitative measure of their biodistributions and metabolic processes by PET/CT imaging, beyond the localization photoacoustic/photothermal (PT) imaging. We first investigated CT contrast performances of TeSe_x_ nano-alloys *in vitro* and *in vivo* with 4T1 tumor-bearing mice with intravenous injection of TS3 (100 μL, 10 mg mL^–1^). TeSe_x_ nano-alloys exhibited a considerable X-ray absorption coefficient of 2.3 HU/mM equal to that of an aqueous iodine standard (iopamidol) which is popularly used clinically at 140 kV of X-ray tube voltage [52] (Supplementary Fig. S19). In addition, superior to the iodine standard, TeSe_x_ nano-alloys have no visible kidney/heart/lung toxicities and longer circulation time, as well as tumor-targeted ability in favor of tumor-targeted therapy. As in Fig. 3e and f, intratumoral CT signal (yellow circles) gradually increased and achieved the maximum at about 8 h post injection, indicating gradual intratumoral accumulation process of TeSe_x_ nano-alloys in accordance with the above-mentioned PA imaging results. Interestingly, we also observed that CT signals in the kidney (red arrows, Supplementary Fig. S21) and bladder (red circles, Supplementary Fig. S21) enhanced with time, indicating that TeSe_x_ nano-alloys could be excreted through the urinary system, possibly owing to their small particle size (about 43 nm, Supplementary Fig. S4). Furthermore, the radionuclide ^64^Cu was facilely labeled to TeSe_x_ nano-alloys (TS3) by a surface coordination method for PET imaging. Supplementary Fig. S22 showed about 90.6% ^64^Cu-labeled efficiency of TeSe_x_ nano-alloys which was measured by instant thin layer chromatography (iTLC). Then, we employed PET to evaluate the *in vivo* delivery and biodistribution of TeSe_x_ nano-alloys. The decay-corrected PET images (Fig. 3g) displayed a high tumor-to-background contrast in the TeSe_x_-^64^Cu treated 4T1 tumor-bearing nude mice. The tumor uptake efficiency of TeSe_x_ nano-alloys was measured by using a quantitative three-dimensional volume-of-interest analysis method. As shown in Fig. 3g–i, the intratumoral accumulation of TeSe_x_ nano-alloys reached the maximum after about 12 h post injection, and TeSe_x_ nano-alloys which were taken up by liver, spleen and kidney were gradually eliminated with time. In Fig. 3g, the metabolic process of TeSe_x_ nano-alloys was also clearly visible (yellow arrows). At 24 h post injection, the mice were sacrificed and major organs were collected for biodistribution study. As shown in Fig. 3j–l, 6.42% ID g^−1^ tumor uptake of TeSe_x_ nano-alloys was achieved at 24 h post injection, and other particles mainly distributed on liver, spleen, kidney, etc. Even though TeSe_x_ nano-alloys widely distributed in the body, their continuous excretion could reduce the potential risk of toxicity. In addition, the biodistributions of TeSe_x_ nano-alloys in major organs were also determined by ICP-OES at the different time points (2, 4, 8, 12 and 24 h) after injection (*n* = 3). As shown in Supplementary Figs S20 and S23, the ICP-OES results more accurately reflect the biodistribution of TeSe_x_ nano-alloys in basic accordance with PET results, and the blood circulation half-time of TS3 was calculated to be about 1.21 h. Nevertheless, TeSe_x_ nano-alloys were confirmed to be an excellent theranostic platform with multimodal PT/PA/CT/PET imaging functions in favor of guiding and monitoring cancer treatment.

### *In vitro* and *in vivo* NIR-II-photothermal therapy performances of TeSe_x_ nano-alloys

**Figure 4.**
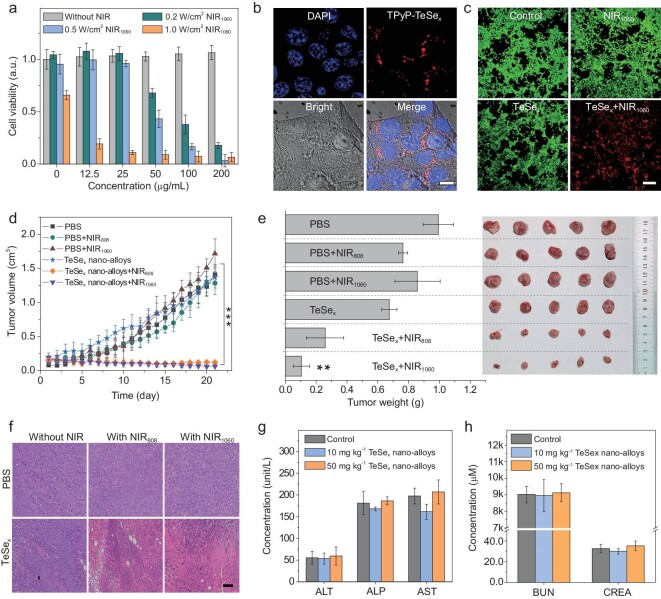
*In vitro* and *in vivo* NIR-II-photothermal therapy performances of TeSe_x_ nano-alloys (TS3). (a) Viability of 4T1 cells after treatment with various concentrations of TeSe_x_ nano-alloys followed by 1060 nm laser irradiation for 5 min at different power densities (0, 0.2, 0.5 and 1.0 W cm^−2^). (b) Cell internalization of TPyP-labeled TeSe_x_ nano-alloys in 4T1 cells (scale bar: 10 μm). (c) Confocal fluorescence images of TeSe_x_ nano-alloys induced photothermal ablation (1.0 W cm^−2^, 5 min, 100 μg mL^−1^) after the 1060 nm laser irradiation (scale bar, 100 μm). (d) Tumor growth of 4T1 tumor-bearing mice with or without laser irradiation after intravenous injection with PBS or 100 μL TeSe_x_ nano-alloys. (e) 4T1 tumor weight comparison after 22-day treatment and the corresponding digital images of extracted tumors (*n* = 5). (f) H&E staining images of tumor tissues (scale bar, 100 μm). Haematological indexes of liver (g) and kidney (h) functions of the mice with intravenous administration of TeSe_x_ nano-alloys. *P* values in (d) and (e) were calculated by two-tailed Student's *t*-test (^***^P < 0.005, ^**^P < 0.01) by comparing with the PBS control group.

Cellular uptake of TeSe_x_ nano-alloys was firstly investigated *in vitro*. TeSe_x_ nano-alloys were facilely labeled with red fluorescent dye 5,10,15,20-tetra(4-pyridyl)-21*H*,23*H*-porphine (TPyP) by virtue of its coordination capability. Confocal fluorescence images of 4T1 cells incubated with TPyP-labeled TeSe_x_ nano-alloys for 1 and 2 h showed that red fluorescence gradually increased inside cells (Fig. 4b and Supplementary Fig. S24), indicating that TeSe_x_ nano-alloys were efficiently internalized into 4T1 cells due to their small size of 43 nm. Thereafter, NIR-II-photothermal cytotoxicity of TeSe_x_ nano-alloys against varied cancer cell lines (4T1, B16, HeLa cells) at different particle concentrations and at different laser power densities were investigated using the standard CCK-8 assay. From Fig. 4a and Supplementary Fig. S25, TeSe_x_ nano-alloys without 1060 nm laser irradiation did not show observable cytotoxicity to all the investigated cancer cells at a concentration up to 200 μg mL^−1^. Under 1060 nm laser irradiation, TeSe_x_ nano-alloys exhibited remarkable concentration- and power-dependent cytotoxicity against various cancer cells (Fig. 4a and Supplementary Fig. S25). Typically, 0.5 W cm^−2^ 1060 nm laser irradiation for 5 min on 100 μg mL^−1^ TeSe_x_ nano-alloys treated cancer cells killed 83.5% 4T1 cells, 98.2% HeLa cells and 81.6% B16 cells. Additionally, green (calcein-AM) and red (propidium iodide) fluorescence staining results also clearly demonstrated high NIR-II-photothermal cytotoxicity of TeSe_x_ nano-alloys (Fig. 4c). In addition, based on obvious absorption and photothermal conversion of TeSe_x_ nano-alloys in the NIR-I window, we also found that TeSe_x_ nano-alloys had remarkable NIR-I-photothermal cytotoxicity against various cancer cells (Supplementary Fig. S27), which can also be an alternative candidate for photothermal therapy of cancer.

Encouraged with the above-confirmed NIR-II-photothermal effects of TeSe_x_ nano-alloys, the NIR-I-/NIR-II-photothermal ablation of solid tumors with TeSe_x_ nano-alloys was further evaluated *in vivo*. Firstly, mice bearing breast 4T1 tumor were randomly divided into six groups (*n* = 5 per group) with approximately the same tumor volume (ca. 100 mm^3^): (i) the PBS group (blank control), (ii) the PBS+NIR808 group, (iii) the PBS+NIR1060 group, (iv) the TeSe_x_ group, (v) the TeSe_x_+NIR808 group, and (vi) the TeSe_x_+NIR1060 group. The NIR treatment group referred to the mice intravenously injected with PBS or TeSe_x_ nano-alloys (100 μL TS3, 10 mg kg^−1^, three times at Day 1, Day 3 and Day 5) and irradiated with 808 nm or 1060 nm laser irradiation (1.0 W cm^−2^ for 5 min) at fixed time points (Day 2, Day 4 and Day 6) after 8 h post injection to ensure sufficient thermal damage to tumor cells. From Fig. 4d, tumor growth was not affected by TeSe_x_ nano-alloys, but significantly suppressed by combination of TeSe_x_ nano-alloys with 1060 nm laser irradiation. The 808 nm laser irradiation plus TeSe_x_ nano-alloys also generated remarkable inhibition effect on tumor growth, but the inhibition efficacy was not as efficient as that of the 1060 nm laser irradiation. After 21-day treatment, tumors were dissected to photograph and weigh as shown in Fig. 4e. The results further demonstrated remarkable inhibition effect of NIR-photothermal TeSe_x_ nano-alloys on tumor growth. Although tumors had not been completely eradicated which possibly resulted from short irradiation time of only 5 min, we felt optimistic because *in vivo* therapeutic efficacy could become better if we extended NIR irradiation time. Furthermore, the hematoxylin-eosin (H&E) staining of major organs and tumor tissues was conducted (Fig. 4f and Supplementary Fig. S28). It was found that NIR-II-photothermal therapy caused significant tumor cell damage. But there was no obvious damage in all major organs after treatment (Supplementary Fig. S28) and no loss in body weight during treatment (Supplementary Fig. S29), suggesting no obvious systematic toxicity of TeSe_x_ nano-alloys. Even at the high injection dose of 50 mg kg^−1^ which was five-fold higher than treatment dose, no damage to liver and kidney functions was visible (Fig. 4g and h). These results indicated that TeSe_x_ nano-alloys were a biocompatible and high-efficacy NIR-photothermal platform.

## CONCLUSION

In conclusion, a series of TeSe_x_ nano-alloys with different ratios of Se/Te and length/diameter were controllably synthesized by the facile co-precipitation method. Incorporating a moderate content of Se (x = 0.43) into the lattice of Te nanostructure effectively eradicated the toxicity of Te, which mainly originated from GSTs up-regulation, and RPL7A/GAPD down-regulation caused subunit organization dysfunction and energy production loss. TeSe_x_ nano-alloys exhibited high NIR-II-photothermal conversion efficiency (77.2%), and had been proved to be a kind of versatile nanotheranostic platform with multiple functions of NIR-II-photothermal therapy and multimodal PT/PA/PET/CT imaging, enabling multimodal imaging-guided NIR-II-photothermal therapy of cancer with high theranostic performances.

## METHODS

The controlled preparation of rod-like TeSe_x_ nano-alloys with different ratios of Te/Se and length/diameter were realized by a facile hydrothermal method, where the Se contents were adjusted by tuning the molar ratio of tellurite to selenite. The comprehensive details, chemicals and characterizations are in the Supplementary data.

## Supplementary Material

nwaa156_Supplemental_File
