# Supplementary material for: Engineering biocompatible TeSex nano-alloys as a versatile theranostic nanoplatform
Source: Natl Sci Rev. 2020 Jul 6;8(6):nwaa156. doi: 10.1093/nsr/nwaa156 (PMC8274553; doi:10.1093/nsr/nwaa156)
Supplement: nwaa156_Supplemental_File [file nwaa156_supplemental_file.doc]

**Supplementary Information**

**Engineering biocompatible TeSe_x_ nano-alloys as a versatile theranostic nanoplatform**

Xiang Ling^1,†^, Zhaokui Jin^2,†^, Qi Jiang^2^, Xiaotao Wang^1^, Bin Wei^3^, Zhongchang Wang^3^, Yangsen Xu^1^, Tianye Cao^2,4^, Jonathan W. Engle^4^, Weibo Cai^4^, Chenliang Su^1^,*, and Qianjun He^2^,*

^1^International Collaborative Laboratory of 2D Materials for Optoelectronic Science & Technoogy, Engineering Technology Research Center for 2D Materials Information Functional Devices and Systems of Guangdong Province, Institute of Microscale Optoeletronics, Shenzhen University, Shenzhen 518060, China;

^2^Guangdong Provincial Key Laboratory of Biomedical Measurements and Ultrasound Imaging, National-Regional Key Technology Engineering Laboratory for Medical Ultrasound, School of Biomedical Engineering, Health Science Center, Shenzhen University, No. 1066 Xueyuan Road, Nanshan District, Shenzhen 518060, China;

^3^Department of Quantum and Energy Materials, International Iberian Nanotechnology Laboratory (INL), Braga 4715-330, Portugal;

^4^Departments of Radiology and Medical Physics, University of Wisconsin-Madison, Madison, WI 53705, USA

**^*^Corresponding authors.** E-mails: nanoflower@126.com; chmsuc@szu.edu.cn

^†^Equally contributed to this work

**Materials**

Sodium tellurite, Tellurium dioxide, Sodium dodecyl sulfate (SDS), hydrazine monohydrate (>98%) and ammonia solution (25-28%) were purchased from Aladdin Ltd (Shanghai). Sodium selenite and PVP360 were commercially obtained from Sigma-Aldrich Ltd. Selenious acid was purchased from TCI Ltd (Shanghai). 4',6-diamidino-2-phenylindole (DAPI), Propidium Iodide, Calcein-AM were purchased from GL biochem Ltd (Shanghai). The CCK-8 kit was purchased from Beyotime Biotechnology Co. Ltd. 5,10,15,20-Tetra(4-pyridyl)-21H,23H-porphine (TpyP) was purchased from J&K Scientific Ltd. All the solvents were used as received without any further purification unless stated otherwise.

**Instruments**

Transmission electron microscope (TEM) images were taken using HT7700 (Hitachi) and Titan Cubed Themis G2 300 (FEI). X-ray diffraction (XRD) patterns were performed on Rigaku Ultima IV using Cu Kα irradiation at a scan rate of 2^o^·min^-1^ with the target voltage at 40 kV and current 40 mA. The UV-Vis absorption spectra were recorded on a UV-3600 UV-Vis-NIR scanning spectrophotometer (Carry 5000, Agilent). The chemical compositions were analysed using X-ray photoelectron spectroscopy (XPS, Thermo Escalab 250, a monochromatic Al Kα X-ray source). All binding energies were referenced to the C 1s peak (284.8 eV) arising from adventitious carbon. The content of Tellurium and Selenium was measured by ICP-OES (Agilent 700).

**Methods**

**Synthesis of Te nano-rods.** Tellurium dioxide (158 mg) and Sodium selenite aqueous solution (0.198 mL, 50 mM) were added into 250 mL round-bottom flask. Then hydrazine monohydrate (100 mL) was added and the formed mixture was stirred at 40^o^C for 30 min. The reaction was terminated by pouring the mixture into SDS aqueous solution (500 mL, 10 mM), followed by centrifugation and washing to obtain the as-formed Te nano-rods.

**Synthesis of TeSe_x_ nano-alloy.** To a 100 mL baker contained H_2_O (50 mL) was added PVP360 (0.5 g). After being completely dissolved, Na_2_TeO_3_ and Na_2_SeO_3_ were added into the mixture. Here, the integral molar quantity of Na_2_TeO_3_ and Na_2_SeO_3_ was 1.5 mmol. Then, N_2_H_4_∙H_2_O (5.0 mL) and NH_3_∙H_2_O (4.5 mL) were added successively to the mixture. The formed mixture was stirred for 30 min and transferred to Teflon-lined stainless-steel autoclave and heated at 100^o^C for 3h. After being cooled to room temperature, ethanol (250 mL) was added into the reaction mixture and centrifuged at 14000 rpm for 45 min. The residue was re-dispersed into 30 mL H_2_O and purified through Millipore Amicon-Ultra-15 (MWCO: 100 kDa). Finally, the TeSe_x_ nano-alloy powder was obtained through the freeze-drying procedure. In this work, four kinds of TeSe_x_ nano-alloy were prepared by employing different molar ratio of Na_2_TeO_3_ to Na_2_SeO_3_ (3:1, 3:2, 1:1 and 2:3 respectively), where the total molar quantity of Na_2_TeO_3_ and Na_2_SeO_3_ was constant (1.5 mmol).

**Photothermal effects of TeSe_x_ nano-alloy**. The photothermal performances of TeSe_x_ nano-alloy were obtained by monitoring the temperature change of sample solutions under the irradiation of NIR laser (808 nm/1060 nm, MW-GX-808/1~5000 mW, MW-IR-1060/1~3000mW, Changchun Laser Optoelectronics Technology Co., Ltd.) at different power densities (0.2-1.0 W cm^-2^). The temperature was recorded by a fixed-mounted thermal imaging camera (FLIR A300-series). The photothermal conversion efficiencies (η) of TeSe_x_ nano-alloy were calculated using the Roper’s method.

***In vitro* cytotoxicity assay.** To evaluate the effect of selenium doping on cytotoxicity *in vitro*, two cell lines (murine breast cancer 4T1 cells as cancer mode, human normal liver L-O2 cells as normal mode) were chosen and purchased from China Type Culture Collection (CTCC) obtained from the American Type Culture Collection (ATCC). The cells were maintained under 5% CO_2_ at 37 °C in Dulbecco’s modified eagle’s medium (DMEM, high glucose, GIBCO) containing 10% fetal bovine serum and 1% penicillin/streptomycin in a humidified incubator. In a cytotoxicity assay, 4T1 cells or L-O2 cells at the density of 1×10^4^ cells were planted in each well of the 96-well plate and cultivated with 100 μL DMEM culture medium per well at 37 °C in a humidified and 5% CO_2_ incubator. After incubation for 24 h, the culture medium was replaced with fresh ones containing the samples (Te, TS1-TS4) at final concentrations of 12.5–200 μg mL^-1^. After another 24 h incubation, CCK-8 assay was used to evaluate the viability of cells (n=5). In a proliferation assay, 4T1 cells at the density of 3×10^3^ cells were planted in a 96-well and the rest steps were similar to the cytotoxicity assay.

***In vivo* toxicity assay.** Animal experiment procedures were in agreement with the guidelines of the Regional Ethics Committee for Animal Experiments and the care regulations approved by the administrative committee on Animal Research in Shenzhen University. Eighteen healthy female BALB/c mice (4 weeks old, ~20 g, purchased from Model Animal Research Center of Nanjing University) were divided into six groups: (1) control group, (2) Te group, (3) TS1 group, (4) TS2 group, (5) TS3 group, (6) TS4 group. Each group was intravenously injected by the corresponding sample at the same dose of 10 and 50 mg kg^-1^. After 7 days feeding, the histological, haematological and blood biochemical indexes were collected.

**RNA-seq analysis.** To evaluate the mechanism of TeSe_x_ nano-alloy on cytotoxicity, 4T1 cell lines were chosen as mode. 4T1 cells at the density of 1×10^5^ cells were planted in each well of the 24-well plate and cultivated with 1 mL DMEM culture medium per well at 37 °C in a humidified and 5% CO_2_ incubator. After incubation for 24 h, the culture medium was replaced with fresh ones containing the samples (Blank, Te, TS1-TS4) at final concentrations of 200 μg mL^-1^. After another 12 h incubation, the cells were collected and washed with PBS quickly, and then added 1 mL of TRIzol reagent per 5×10^5^ cells to form a clear and viscous solution. The final solution was stored in liquid nitrogen to waiting for subsequent RNA extraction. A total amount of 3 μg RNA per sample was used as input material for the RNA sample preparations. Sequencing libraries were generated using NEBNext® UltraTM RNA Library Prep Kit for Illumina® (NEB, USA) following manufacturer’s recommendations and index codes were added to attribute sequences to each sample. The clustering of the index-coded samples was performed on a cBot Cluster Generation System using TruSeq PE Cluster Kit v3-cBot-HS (Illumia) according to the manufacturer’s instructions. After cluster generation, the library preparations were sequenced on an Illumina Hiseq platform and 125 bp/150 bp paired-end reads were generated. Differential expression analysis of two conditions/groups (two biological replicates per condition) was performed using the DESeq2 R package (1.16.1). The resulting P-values were adjusted using the Benjamini and Hochberg’s approach for controlling the false discovery rate. Genes with an adjusted P-value <0.05 found by DESeq2 were assigned as differentially expressed. Gene Ontology (GO) enrichment analysis of differentially expressed genes was implemented by the clusterProfiler R package, in which gene length bias was corrected. GO terms with corrected P value less than 0.05 were considered significantly enriched by differential expressed genes. KEGG is a database resource for understanding high-level functions and utilities of the biological system, such as the cell, the organism and the ecosystem, from molecular-level information, especially large-scale molecular datasets generated by genome sequencing and other high-through put experimental technologies (http://www.genome.jp/kegg/). We used clusterProfiler R package to test the statistical enrichment of differential expression genes in KEGG pathways.

***In vitro* photothermal ablation of cancer cells.** The 4T1 cells, B16 melanoma cells and human HeLa cervical carcinoma cells were purchased from China Type Culture Collection (CTCC) obtained from the American Type Culture Collection (ATCC). Cells were routinely tested for mycoplasma contamination using MycoSET Mycoplasma real-time PCR detection Kit (Life Technologies, Foster City, CA, USA). 4T1 or B16, HeLa cells were first seeded in 96-well plates at a density of 1×10^4^ cells per well with a DMEM culture medium containing 10% fetal bovine serum at 37°C in a humidified and 5% CO_2_ incubator. After 24 h incubation, the culture medium was replaced by the fresh medium with TS3 dispersion at different concentration (12.5–200 μg mL^-1^). After another 4 h of incubation, the culture was removed and washed with PBS three times, and fresh DMEM medium was added into the wells. Then, the wells were irradiated with 808 nm or 1060 nm NIR laser at varied power density (1.0 W cm^-2^, 0.5 W cm^-2^, 0.2 W cm^-2^) for 5 min per well. After another incubation of 24 h, 10 μL CCK-8 solution was added into each well and incubated for 0.5 h, the plate was detected by the Bio-Tek multi-mode microplate reader at 450 nm. The cytotoxicity was expressed as the percentage of cell viability as compared with the blank control. Each data point was represented as a mean ± standard deviation of five independent experiments (*n* =5).

**Confocal Fluorescence Imaging.** 4T1 cells with a density of 1×10^5^ cells were seeded into a CLSM-special cell-culture dish, which were cultured at 37°C under 5% CO_2_ atmosphere for 12 h incubation. Then, 200 μL of TS3 DMEM solution (100 μg mL^-1^) was added into the dish. After co-incubation for 4 h, the culture was removed and washed with PBS three times, and fresh DMEM medium was added into the wells. Then, the cell solution was irradiated by an 808 nm or 1060 nm laser for 5 min at a power density of 1.0 W cm^−2^. After further incubation of 12 h, confocal fluorescence images of calcine AM (green) and PI (red) co-stained cells further confirmed the death of 4T1 cells induced by TS3 after laser irradiation. To investigate uptake of TS3 by cells, a typical fluorescent dye, 5,10,15,20-Tetra(4-pyridyl) porphyrin (TPyP) were firstly loading by TS3 *via* impregnation method to obtain TS3-TPyP. Then, 4T1 cells were seeded into a CLSM-special cell-culture dish, and co-incubated with TS3 (100 μg mL^-1^) for 1, 2, 4 h. After cell fixation and 3-4 times wash with PBS, the cells were stain with DAPI (50 μg mL^-1^) for 15 min, and observed with CSLM.

***In vivo* tumor therapy**. All healthy female BALB/c mice (4 weeks old) were purchased from Model Animal Research Center of Nanjing University and all the *in vivo* experiments followed the protocols approved by the Animal Care and Use Committee of the Shenzhen University. To develop the tumor model, 4T1 cells (1×10^6^ cells/site) suspended in 100 μL PBS solution were injected into the hind limb of mice. While the tumor size reached approximately 120-150 mm^3^ (designed as Day 0), the treatment was performed. Two experienced researchers randomly divided the mice into six groups (n = 5 per group): (1) blank control group that intravenously injected with 100 μL PBS without laser irradiation, (2) control group that intravenously injected with 100 μL PBS with 808 nm laser irradiation for 5 min, (3) control group that intravenously injected with 100 μL PBS with 1060 nm laser irradiation for 5 min, (4) TS3 group that intravenously injected with TS3 at dosage of 10 mg kg^-1^ without laser irradiation, (5) Photothermal therapy group that intravenously injected with TS3 at dosage of 10 mg kg^-1^ with 808 nm laser irradiation for 5 min, (6) Photothermal therapy group that intravenously injected with TS3 at dosage of 10 mg Kg^-1^ with 1060 nm laser irradiation for 5 min. All mice were anesthetized before NIR laser irradiation. The intravenous injection was administrated on Day 1, 3, 5. Group 2, 5 and 6 were treated with 808 nm or 1060 nm laser at 1.0 W cm^-2^ for 5 min at tumor sites after 8 h injection on Day 2,4,6. The body weight and tumor volume (V = (ab^2^)/2, where a and b refer to the largest length and width of tumor, respectively) of each mouse were recorded every other day. The mice were humanely killed after 21 days of treatment and all the tumors were collected.

**Histological stained analysis**. All mice were humanely killed and main organs (heart, liver, spleeny, lung and kidney) and tumors were harvested after 21 days treatment, fixed in a 4% polyoxymethylene solution, and embedded in paraffin for H&E staining.

***In vivo* photothermal imaging (PTI).** The 4T1 tumor-bearing mice model was established by injecting 4T1 cells at a density of 1×10^6^/mL into the hind limb of each Balb/c mouse (~20 g, purchased from Model Animal Research Center of Nanjing University). After the mean volume of the tumors reached about 100 mm^3^, two experienced researchers randomly divided the mice into two groups (*n* = 3 per group). The tumor-bearing mice were injected with 100 μL of PBS (Group 1), or 10 mg kg^-1^ TS3 (Group 2) through the vein of tail. After 8 h injection, the mice were anesthetized by 4% chloral hydrate (120 μL) and then irradiated with the 1060 nm laser at 1 W cm^-2^ for 5 min. During the course of irradiation, the temperature changes were recorded by the infrared thermal imaging cameras (FLIR A300-series). Group 1 was taken as the control.

***In vivo* photoacoustic imaging (PAI).** All mouse imaging experiments were performed using a real-time multispectral optoacoustic tomographic imaging system (Vevo 2100 LAZR system, Visual Sonic Inc. New York, NY) equipped with a 40 MHz, 256-element linear array transducer on tumors. For *in vitro* PA imaging, different wavelengths (from 680 to 960 nm) of excitation light were used to collect the photoacoustic signals, while provided the following PA imaging test with an excitation light of 805 nm. Different concentrations of TS3 (0.25, 0.5, 1.0, 2.0 mg mL^-1^) solution were used to evaluate the linearity of the PA signal. For *in vivo* PA imaging study, subcutaneous 4T1 tumor bearing Balb/c mice were anesthetized by 1% isoflurane delivered *via* a nose cone, and then the TS3 (100 μL, 2 mg mL^-1^) were injected *via* the tail vein. *In vivo* PA images were acquired before injection and at different time points post injection (2 h, 4 h, 8 h, 12 h and 24 h) using the multispectral optoacoustic tomography system at a wavelength of 810 nm. A region of interest (ROI) volume consisting of transverse slices with a step size of 0.4 mm, spanning through the tumor region, was selected by manual inspection of live MSOT images. The average PA signal of the tumor area was extracted using the multispectral optoacoustic tomography software.

***In vivo* computed tomography (CT) imaging**. Balb/c 4T1-bearing tumors mice were anesthetized with 1% isoflurane. The mouse was intravenously injected with TS3 (100 μL, 10 mg mL^−1^) (n=3). In vivo CT scanning was performed after injection of the TS3 at different time points (0 h, 2h, 4 h, 8 h, 12 h, 24 h). All CT scans were performed using the PET/CT system (G8, Perkinelmer).

***In vivo* Positron Emission Computed Tomography (PET) imaging.** To evaluate the PET imaging property, the ^64^Cu-labeling of TS3 was firstly carried out. ^64^Cu was produced with an onsite cyclotron (GE PETrace) at the University of Wisconsin-Madison. ^64^CuCl_2_ (150 MBq) was diluted in 0.1 M sodium acetate buffer (pH 5.5) and mixed with 200 µL of PEG-SH and 100 µL of TS3. The reaction was conducted at 65 °C for 1 h with constant shaking. TLC determined the labeling yield at different time points using 50 mM EDTA solution as the mobile phase. Fluorescence imaging was performed by using an IVIS Spectrum Preclinical. The resulting product was purified using a PD-10 column with PBS as the mobile phase. For normal PET imaging, 4T1 tumor bearing BALB/c mice were injected with ^64^Cu-TS3 via the tail vein before serial PET scans. Quantitative PET data was presented as a percentage of the injected dose per gram (%ID/g). For biodistribution studies, major organs were collected and wet-weighed at designed time points p.i. The radioactivity uptake by the tissue was measured by using a gammacounter (Perkin-Elmer) and presented as %ID/g (mean ± SD).

**Table S1** The composition of Te NRs, TS1, TS2, TS3 and TS4 measured by ICP.

| **Name** | **Se/Te**  **mass ratio** | **Se/Te**  **molar ratio** | **Chemical structures** |
| --- | --- | --- | --- |
| Te NRs | 0.0017 | 0.0028 | Te_99.72_ |
| TS1 | 0.13 | 0.21 | Te_0.82_Se_0.18_ |
| TS2 | 0.21 | 0.33 | Te_0.75_Se_0.25_ |
| TS3 | 0.26 | 0.43 | Te_0.7_Se_0.3_ |
| TS4 | 0.30 | 0.48 | Te_0.67_Se_0.33_ |


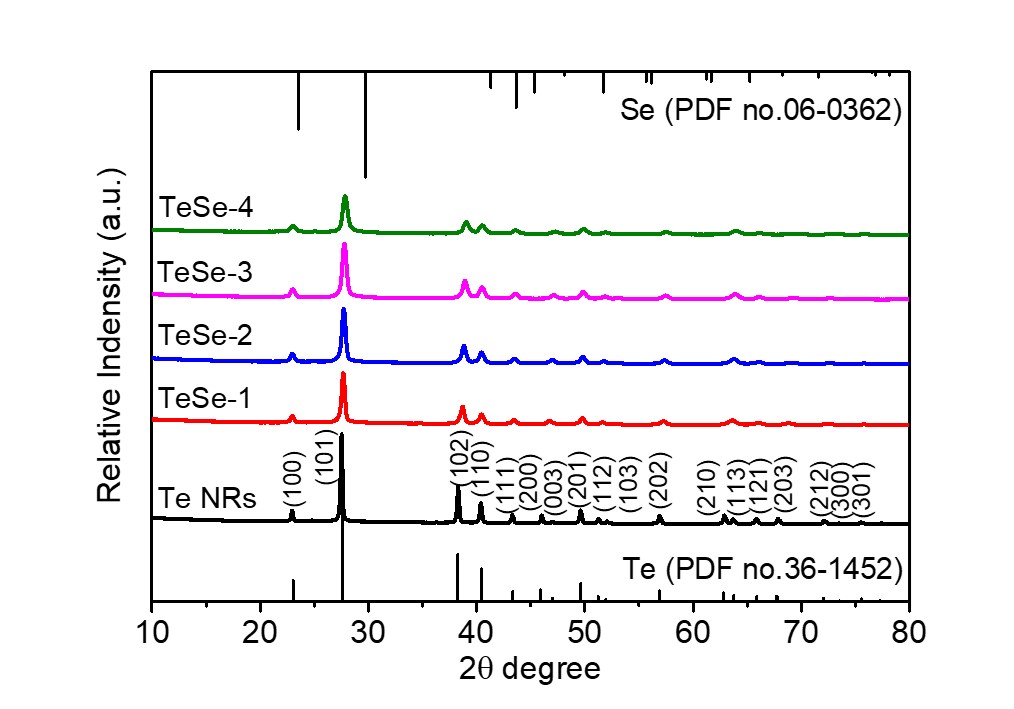


**Figure S1.** XRD patterns of Te NRs, TS1, TS2, TS3 and TS4.

**Figure S2.** Observed, calculated and difference profiles obtained from the Rietveld refinement of Te, TS1, TS2 and TS4 using P3_1_21 space group with hexagonal unit cell.

**Table S2** Surface Se/Te molar ratio of TS1, TS2, TS3 and TS4 obtained from XPS.

| **Name** | **Surface Se/Te molar ratio** |
| --- | --- |
| TS1 | 0.94 |
| TS2 | 1.16 |
| TS3 | 1.33 |
| TS4 | 1.5 |
| TS3 (etching 1 min) | 0.39 |
| TS3 (etching 2 min) | 0.35 |

**Figure S3.** Survey, Te 3d and Se 3d XPS patterns of Te NRs, TS1, TS2, TS3 and TS4.

**Figure S4.** TEM images and size distribution (inset) of Te NRs, TS1, TS2, TS3 and TS4, scale bar: 100 nm.

**Table S3.** Reitveld refined position coordinates and lattice parameters of Te NRs, TS1, TS2, TS3 and TS4 in P3_1_21 space group.

| **Name** | **Lattice parameters** | | | | | **Fractional** | **Coordinates** | |  | **Statistical parameter** | |
| --- | --- | --- | --- | --- | --- | --- | --- | --- | --- | --- | --- |
|  | **a=b (Å)** | **c (Å)** | **c/a** | **a=b (^o^)** | **g (^o^)** | **x** | **y** | **z** | | **R_p_** | **R_wp_** |
| Te NRs | 4.455 | 5.903 | 1.325 | 90 | 120 | -0.2636 | 0 | 1/3 | | 5.538 | 7.418 |
| TS1 | 4.450 | 5.822 | 1.308 | 90 | 120 | -0.2627 | 0 | 1/3 | | 3.075 | 4.36 |
| TS2 | 4.449 | 5.792 | 1.302 | 90 | 120 | -0.2612 | 0 | 1/3 | | 3.038 | 4.715 |
| TS3 | 4.448 | 5.772 | 1.298 | 90 | 120 | -0.2610 | 0 | 1/3 | | 3.167 | 4.023 |
| TS4 | 4.452 | 5.759 | 1.294 | 90 | 120 | -0.2578 | 0 | 1/3 | | 3.635 | 4.128 |

**Table S4. The stability of Te and TeSe_x_ nano-rods measured by ICP.**

| Name | The amount of released Te (mg/L) |
| --- | --- |
| Te | 14.088 |
| TS1 | 9.006 |
| TS2 | 6.491 |
| TS3 | 3.615 |

Note: The Te and TeSe_x_ nano-rods were stored in water (1000 mg/L) for 8 month. The release of Te from various samples was measured with the filtered solution through Millipore Amicon-Ultra-15 (MWCO: 100 kDa).

**
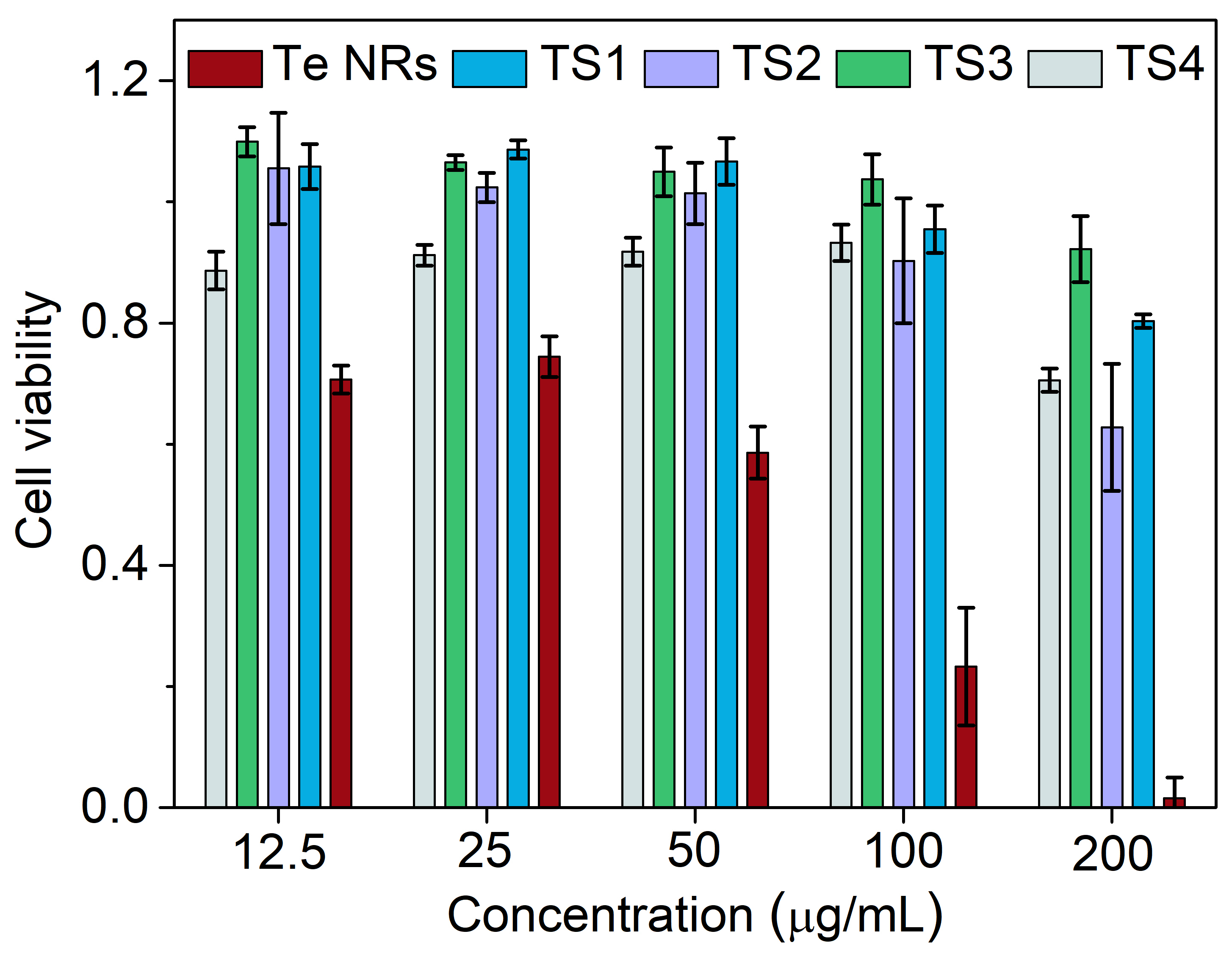
**

**Figure S5.** Relative viabilities of normal cells line LO2 after treatment with varied concentrations of TeSe_x_ nano-alloys.

**Figure S6.** Venn diagram showing results of the differentially expressed genes, a) upregulated, b) downregulated. T1-T5 are on behalf of the Te NRs, TS1, TS2, TS3, TS4, respectively. T0 is blank control.

**a**





**b**





**c**





**d**





**e**





**Figure S7.** (a-e) Volcano plots of significantly differentially expressed genes. T1-T5 are on behalf of the Te NRs, TS1, TS2, TS3, TS4, respectively. T0 is blank control.


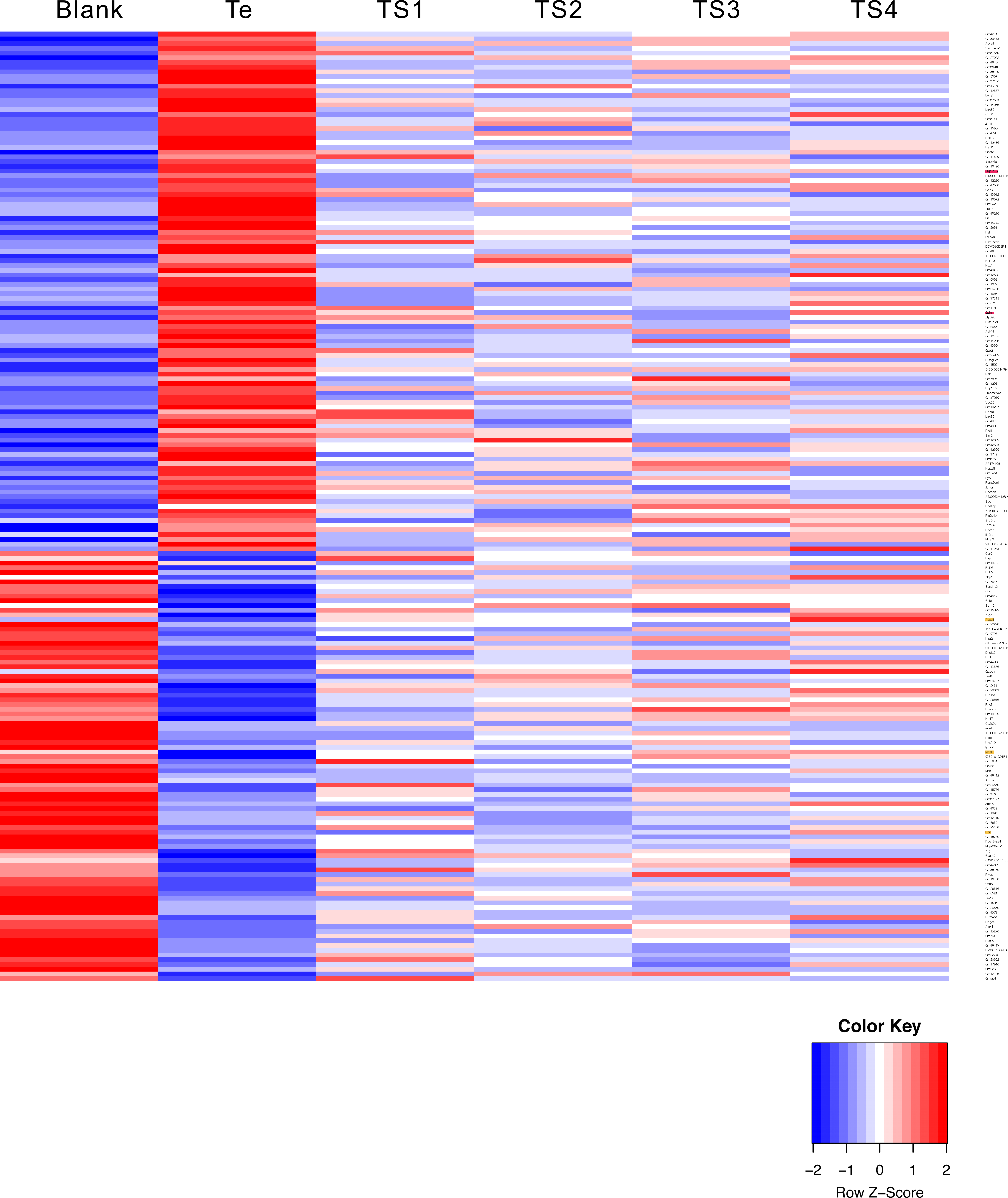


**Figure S8.** Heatmap showing the differentially expressed genes among six samples.

**Table S5.** Up-regulated genes related with functions and phenotypes.

| **Term** | **Gene symbols** | **P value** | | | | |
| --- | --- | --- | --- | --- | --- | --- |
|  |  | Te vs Blank | TS1 vs Blank | TS2 vs Blank | TS3 vs Blank | TS4 vs Blank |
| Glutathione metabolism | Gpx2;Gsta1 | 0.008191 | 0.00408 |  |  | 0.01733 |
| Metabolism of xenobiotics by cytochrome P450 | Cyp3a13;Gsta1 | 0.008191 |  |  |  |  |
| Drug metabolism-cytochrome P450 | Cyp3a13;Gsta1 | 0.008191 |  |  |  |  |
| Dorso-ventral axis formation | Piwil4 |  | 0.03913 | 0.03272 |  |  |
| Nitrogen metabolism | Hal |  | 0.04088 | 0.03418 |  |  |
| Histidine metabolism | Hal |  | 0.0461 | 0.03564 |  |  |
| alpha-Linolenic acid metabolism | Plb1 |  |  | 0.02978 |  |  |
| Protein export | Immp2l |  |  | 0.04727 |  |  |
| ABC transporters | Abca4;Abcb6 |  |  |  | 0.001959 |  |
| Osteoclast differentiation | Fcgr1;Pirb |  |  |  | 0.01288 |  |
| Glycolysis / Gluconeogenesis | Gapdh;Gapdh-ps15 |  |  |  |  | 0.02181 |
| Cyanoamino acid metabolism | Ggt7 |  |  |  |  | 0.02242 |
| Taurine and hypotaurine metabolism | Ggt7 |  |  |  |  | 0.0371 |
| Arachidonic acid metabolism | Alox12b;Ggt7 |  |  |  |  | 0.04468 |

**Table S6.** Down-regulated genes related with functions and phenotypes.

| **Term** | **Gene symbols** | **P value** | | | | |
| --- | --- | --- | --- | --- | --- | --- |
|  |  | Te vs Blank | TS1 vs Blank | TS2 vs Blank | TS3 vs Blank | TS4 vs Blank |
| Glycolysis / Gluconeogenesis | Gapdh;Gapdh-ps15 | 0.008097 |  |  |  |  |
| Ribosome | Rpl26;Rpl7a | 0.02791 |  | 0.02121 |  | 0.008067 |
| Systemic lupus erythematosus | Hist1h4j;Hist3h2a;Hist1h2ak |  | 0.001189 |  |  |  |
| Phenylalanine, tyrosine and tryptophan biosynthesis | Il4i1;Gm21948 |  |  | 0.00007414 |  |  |
| Phenylalanine metabolism | Il4i1;Gm21948 |  |  | 0.0005336 |  |  |
| Alanine, aspartate and glutamate metabolism | Il4i1;Gm21948 |  |  | 0.001811 |  |  |
| Tyrosine metabolism | Il4i1;Gm21948 |  |  | 0.002274 |  |  |
| Cysteine and methionine metabolism | Il4i1;Gm21948 |  |  | 0.002525 |  |  |
| Tryptophan metabolism | Il4i1;Gm21948 |  |  | 0.003351 |  |  |
|  |  |  |  |  |  |  |
| Valine, leucine and isoleucine degradation | Alox12b;Ggt7 |  |  | 0.004124 |  |  |
| Phagosome | Mrc2;Cd209c |  |  | 0.04511 |  |  |
| Chemokine signaling pathway | Gng7;Gng13 |  |  | 0.04937 |  |  |
| Purine metabolism | Entpd4;Adcy5 |  |  |  | 0.03566 |  |
| Alzheimer's disease | Gapdh;Gapdh-ps15 |  |  |  | 0.04047 |  |

**a**

**b**

**c**

**Figure S9.** Significantly upregulated metabolic pathway in group of Te compared to blank. (a) Metabolism of xenobiotics by cytochrome P450, (b) Glutathione metabolism. (c) Glycolysis.

**Figure S10.** Effects of TeSe_x_ nano-alloys on the expression levels of GAPD, RPL7A and GSTs proteins by Western blotting analysis. *P* values were calculated by two-tailed Student’s *t*-test (****P*<0.005, ***P*<0.01, **P*<0.05) by comparing with the control group.

**Figure S 11.** Intracellular GSH levels before and after treatment with TeSe_x_ nano-alloys**.**

**Figure S12.** (a) Photothermal effect of an aqueous dispersion of TeSe_x_ under the irradiation with 1060 nm laser for certain periods, and then the laser was switched off. (b-f) The linear relationship between time and −lnθ calculated from cooling period after the laser was turned off of different samples (b, Te NRs; c, TS1; d, TS2; e, TS3; f, TS4).

**Figure S13.** Photothermal conversion stability of TS3 for five 1060-laser on/off cycles (1.0 W cm^−2^).

**Figure S14.** (a) NIR-I-photothermal curves of TS3 solutions at various concentrations under irradiation of 808 nm laser at a laser power density of 1 W cm^−2^, (b) the linear relationship between time and −lnθ calculated from cooling period after the laser was turned off, and (c) photothermal effect of TS3 (100 μg/mL) under NIR808 irradiation at different power densities.


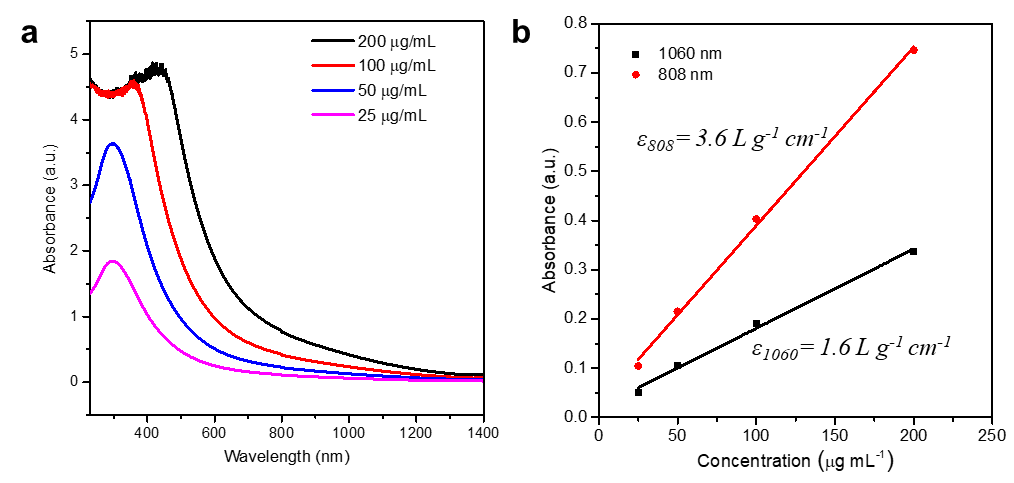


**Figure S15.** Absorption spectra of TS3 with different concentrations and the calculated extinction coefficient.


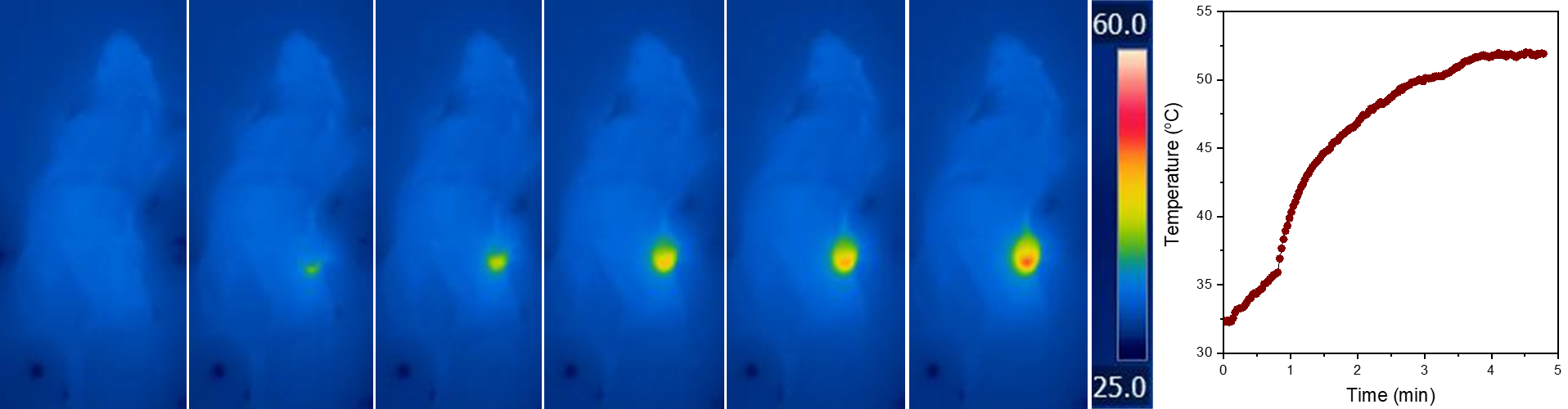


**Figure S16.** *In vivo* photothermal imaging tracking of one 4T1 tumor-bearing mouse under the 808 nm laser irradiation after intravenous injection with TS3 nano-alloys, and the temperature change at the tumor site.

**Figure S17.** The detection of singlet oxygen using ABDA after TS3 nano-alloys were irradiated with 1060 nm laser for different time periods.

**Figure S18.** *In vitro* PA signal of TS3. (a) the dependence of PA imaging signal of TS3 solution on its concentration, (b) the corresponding quantitative stastical analysis of Figure a.

**Figure S19.** *In vitro* CT signal of TS3. (a) the dependence of CT imaging signal of TS3 solution on its concentration, (b) the corresponding quantitative stastical analysis of Figure a.

**Figure S20.** Blood circulation lifetime of TeSex nano-alloys after intravenous injection into mice (n = 3).

**Figure S21.** *In vivo* CT signal of TS3 in kidney (red arrow) and bladder (red circle).

**Figure S22.** In vitro PET signal of ^64^Cu-TS3. (a) the labeling yield at different time points by TLC, (b) the corresponding quantitative stastical analysis of Figure a.

**Figure S23.** TS-3 biodistribution in 4T1 tumor-bearing mice measured by ICP data at the different time points (2, 4, 8, 12 and 24 h) post injection (*n* = 3). Mean value and error bar are defined as mean and s.d., respectively.


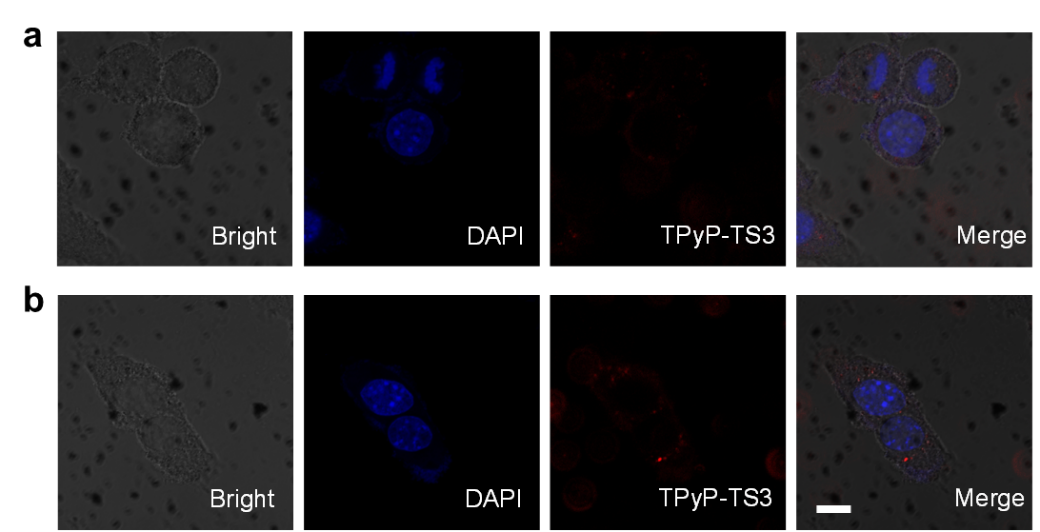


**Figure S24.** Cell internalization of TPyP-TS3 towards 4T1 cells after (a) 1 h and (b) 2 h incubation (Scale bar, 10 μm).

**Figure S25.** Relative viabilities of different cancer cell lines after treatment with varied concentrations of TS3 at different 1060 nm laser power densities (0, 0.5, 1.0 W cm^−2^).

**Figure S26.** Relative viabilities of different cancer cell lines after treatment with varied concentrations of TS3 at different 808 nm laser power densities (0, 0.5, 1.0 W cm^−2^).

**Figure S27.** 4T1 cells stained with calcein AM (live cells, green fluorescence) and PI (dead cells, red fluorescence) after different treatments (scale bar: 200 nm).

**Figure S28.** H&E staining for pathological changes in organ tissues from each group to reveal the effectiveness of in vivo photothermal therapy (scale bar, 200 μm).

**Figure S29.** Body-weight changes in different groups during 21 days treatment
